# *catena*-Poly[[(diaqua­calcium)-bis­(μ-2-fluorobenzoato)-1′:1κ^3^ *O*:*O*,*O*′;1:1′′κ^3^ *O*,*O*′:*O*] 2,2′-bipyridine hemi­solvate]

**DOI:** 10.1107/S1600536811053311

**Published:** 2011-12-17

**Authors:** Bi-Song Zhang, Jian-Li Lin, Xiu-Fang Jin, Rong Huang, Ling-Ling Luo

**Affiliations:** a College of Material Science and Chemical Engineering, Jinhua College of Profession and Technology, Jinhua, Zhejiang 321017, People’s Republic of China; b State Key Laboratory Base of Novel Functional Materials and Preparation, Science Center of Applied Solid State Chemistry Research, Ningbo University, Ningbo, Zhejiang 315211, People’s Republic of China

## Abstract

In the title compound, {[Ca(C_7_H_4_FO_2_)_2_(H_2_O)_2_]·0.5C_10_H_8_N_2_}_*n*_, the Ca^II^ atom is coordinated by eigth O atoms from four 2-fluoro­benzoate ligands and two water mol­ecules, resulting in a distorted CaO_8_ square-anti­prismatic coordination environment. The 2-fluoro­benzoate ligand bridges two symmetry-related Ca^II^ atoms, giving rise to a chain structure extending along [100]. The distances between the Ca atom and its two symmetry-related counterparts are 4.054 (2) and 4.106 (2) Å. The polymeric chains are connected by classical O—H⋯N hydrogen bonds into a layer structure parallel to (010). The layers are connected by non-classical C—H⋯F hydrogen bonds into a three-dimensional supra­molecular structure. O—H⋯O and C—H⋯O inter­actions also occur. The uncoordinated 2,2′-bipyridine mol­ecule is located on a centre of symmetry at the mid-point of the bond between the two heterocycles. One of the two benzene rings is disordered over two sites with occupancy factors of 0.60 and 0.40.

## Related literature

For other metal complexes with the 2-fluoro­benzoato ligand, see: Zhang *et al.* (2005*a* ▶,*b* ▶); Zhang (2006 ▶, 2008) ▶; Jin (2011 ▶). For related structures, see: Zhang (2009 ▶); Karipides *et al.* (1988 ▶).
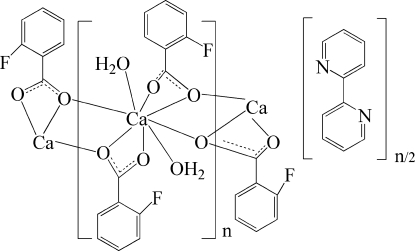


## Experimental

### 

#### Crystal data

[Ca(C_7_H_4_FO_2_)_2_(H_2_O)_2_]·0.5C_10_H_8_N_2_
*M* *_r_* = 432.41
Triclinic, 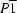

*a* = 7.9063 (16) Å
*b* = 10.212 (2) Å
*c* = 12.147 (2) Å
α = 94.68 (3)°
β = 104.33 (3)°
γ = 92.98 (3)°
*V* = 944.4 (3) Å^3^
*Z* = 2
Mo *K*α radiation
μ = 0.39 mm^−1^
*T* = 295 K
0.34 × 0.19 × 0.16 mm

#### Data collection

Rigaku R-AXIS RAPID diffractometer
Absorption correction: multi-scan (*ABSCOR*; Higashi, 1995 ▶) *T*_min_ = 0.914, *T*_max_ = 0.939
7478 measured reflections
3306 independent reflections
2136 reflections with *I* > 2σ(*I*)
*R*_int_ = 0.053

#### Refinement

*R*[*F* ^2^ > 2σ(*F* ^2^)] = 0.061
*wR*(*F* ^2^) = 0.214
*S* = 1.17
3306 reflections
271 parameters
H-atom parameters constrained
Δρ_max_ = 0.64 e Å^−3^
Δρ_min_ = −0.73 e Å^−3^

### 

Data collection: *RAPID-AUTO* (Rigaku, 1998 ▶); cell refinement: *RAPID-AUTO*; data reduction: *CrystalStructure* (Rigaku/MSC, 2002 ▶); program(s) used to solve structure: *SHELXS97* (Sheldrick, 2008 ▶); program(s) used to refine structure: *SHELXL97* (Sheldrick, 2008 ▶); molecular graphics: *SHELXTL* (Sheldrick, 2008 ▶); software used to prepare material for publication: *SHELXL97*.

## Supplementary Material

Crystal structure: contains datablock(s) I, global. DOI: 10.1107/S1600536811053311/rk2307sup1.cif

Structure factors: contains datablock(s) I. DOI: 10.1107/S1600536811053311/rk2307Isup2.hkl

Additional supplementary materials:  crystallographic information; 3D view; checkCIF report

## Figures and Tables

**Table 1:** Hydrogen-bond geometry (Å, °)

*D*—H⋯*A*	*D*—H	H⋯*A*	*D*⋯*A*	*D*—H⋯*A*
O1—H1*A*⋯N1	0.82	2.21	2.835 (5)	133
O1—H1*B*⋯O5^i^	0.82	2.02	2.782 (5)	154
O2—H2*A*⋯O4^ii^	0.82	2.12	2.741 (5)	132
O2—H2*B*⋯F1^iii^	0.82	2.62	3.363 (5)	151
C7—H7⋯O2^i^	0.93	2.60	3.189 (7)	122
C10—H10⋯F2^iv^	0.93	2.54	3.281 (2)	136

Symmetry codes: (i) 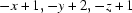; (ii) 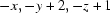; (iii) 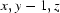; (iv) 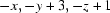.

**Fig. 1.**
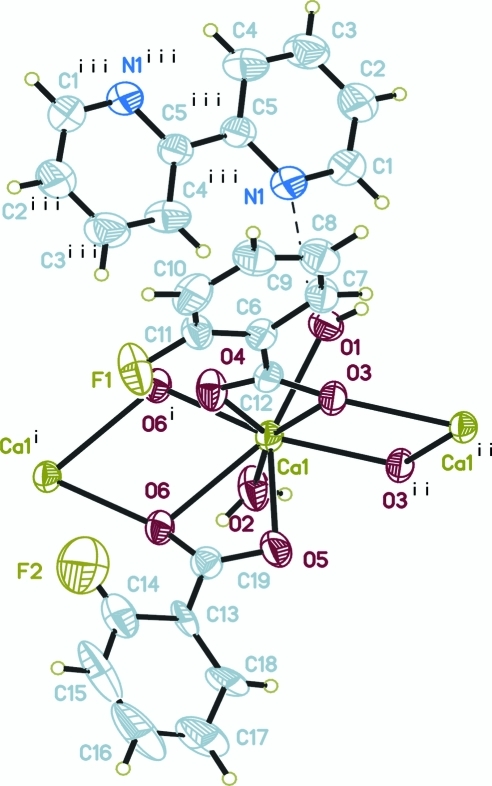
The structure unit of the title compound, showing the atom-labelling scheme. Displacement ellipsoids are drawn at the 50% probability level. Symmetry codes: (i) -x, 2-y, 1-z; (ii) 1-x, 2-y, 1-z); (iii) -x, 2-y, -z.

**Fig. 2.**
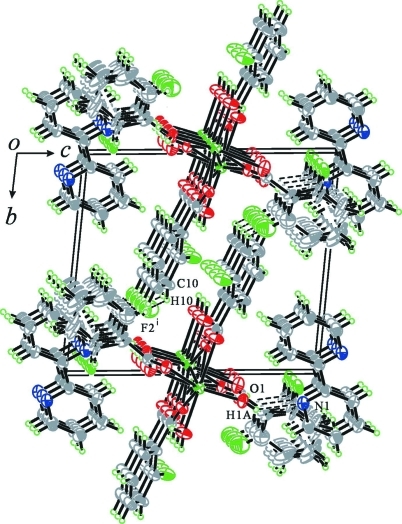
A packing diagram of the title complex, viewed along the a axis. The O1–H1A···N1 and C10–H10···F2v hydrogen bonds (dashed lines) in the title compound. Symmetry code: (v) -x, 3-y, 1-z.

## supplementary crystallographic information

### Comment

A metal ions with 2-fluorobenzoato ligands can form, among others, mononuclear, dinuclear, one-dimensional chain complexes (Zhang *et al.*, 2005**a*, b*; Zhang, 2006, 2008; Jin, 2011). Only a few reports of one-dimensional chain structure complexes includinng uncoordinated 2,2'-bipyridine molecules have been published.

In this paper we would like to report the synthesis, molecular and crystal structures of an one-dimensional chain complex including 2-fluorobenzoato, 2,2'-bipyridine and calcium(II). The crystal structure of title compound is similar to previously published structures (Zhang, 2009; Karipides *et al.*, 1988). Within the title compound, each Ca^II^ ion is coordinated by eight O atoms from two water molecules and four carboxyl groups of 2-fluorobenzoic acid anions in a distorted square-antiprismayic geometry. Two µ_3_-carboxyl group of the 2-fluorobenzoic anions bridges two symmetry related calcium(II) atoms giving rise to an one-dimensional chain structure extending along the [1 0 0] direction, with Ca–O bond lengths in the range of 2.381 (4)Å to 2.726 (4)Å. Separation between Ca1 and Ca1^i^, Ca1 and Ca1^ii^ [symmetry codes: (i) -*x*, 2-*y*, 1-*z*; (ii) 1-*x*, 2-*y*, 1-*z*] are 4.054 (2)Å and 4.106 (2)Å (Fig. 1). The polymeric chains are connected *via* O1–H1A···N1 hydrogen bonds interactions between the coordinates water and 2,2'-bipyridine molecules in to a two dimensional layer structure parallel to (0 1 0) (Fig.2). The middle of bond C5–C5^iii^ [symmetry code: (iii) -*x*, 2-*y*, -*z*] of non-coordinated 2,2'-bipyridine molecule is located in center of symmetry position. The layers are connected by C10–H10···F2^v^ [symmetry code: (v) -*x*, 3-*y*, 1-*z*] weak hydrogen bonds interactions in to a three-dimensional supramolecular structure (Fig. 2). The  O1–H1A···N1 and C10–H10···F2^v^ bond lengths are 2.835 (5)Å and 3.281 (2)Å, the O1–H1A···N1 and C10–H10···F2^v^ bond angles are 133° and 136°.

### Experimental

CaCO_3_ (0.1005 g, 1.00 mmol), 2-fluorobenzoic acid (0.0625 g, 0.45 mmol), 2,2'-bipyridine (0.0512 g, 0.33 mmol), CH_3_OH/H_2_O (v/v = 1:2, 15 ml) were mixed and stirred for 2.0 h. Subsequently, the resulting cream suspension was heated in a 23 ml Teflon-lined stainless steel autoclave at 433 K for 5800 minutes. After the autoclave was cooled to room temperature according to the procedure at 2600 minutes. The solid was filtered off. The resulting filtrate was allowed to stand at room temperature, and slow evaporation for 2 months afforded colorless block single crystals.

### Refinement

A C-bound H atoms were placed in calculated positions, with C–H = 0.93Å and *U*_iso_(H) = 1.2*U*_eq_(C), and were refined using the riding-model approximation. The H atoms of the water molecule were located in a difference Fourier map and refined with an O–H distance restraint of 0.82Å and *U*_iso_(H) = 1.5*U*_eq_(O). One - F2 atom and benzene ring (C13-C18) are disordered over their sites with occupancy factors of 0.60 and 0.40.

### Crystal data

**Table:**

[Ca(C_7_H_4_FO_2_)_2_(H_2_O)_2_]·0.5C_10_H_8_N_2_	*Z* = 2
*M**_r_* = 432.41	*F*(000) = 446
Triclinic, *P*1	*D*_x_ = 1.521 Mg m^−^^3^
Hall symbol: -P 1	Mo *K*α radiation, λ = 0.71073 Å
*a* = 7.9063 (16) Å	Cell parameters from 5423 reflections
*b* = 10.212 (2) Å	θ = 3.2–25.0°
*c* = 12.147 (2) Å	µ = 0.39 mm^−^^1^
α = 94.68 (3)°	*T* = 295 K
β = 104.33 (3)°	Block, colourless
γ = 92.98 (3)°	0.34 × 0.19 × 0.16 mm
*V* = 944.4 (3) Å^3^	

### Data collection

**Table:**

Rigaku R-AXIS RAPID diffractometer	3306 independent reflections
Radiation source: fine-focus sealed tube	2136 reflections with *I* > 2σ(*I*)
graphite	*R*_int_ = 0.053
ω scans	θ_max_ = 25.0°, θ_min_ = 3.2°
Absorption correction: multi-scan (*ABSCOR*; Higashi, 1995)	*h* = −9→9
*T*_min_ = 0.914, *T*_max_ = 0.939	*k* = −12→12
7478 measured reflections	*l* = −14→14

### Refinement

**Table:**

Refinement on *F*^2^	Primary atom site location: structure-invariant direct methods
Least-squares matrix: full	Secondary atom site location: difference Fourier map
*R*[*F*^2^ > 2σ(*F*^2^)] = 0.061	Hydrogen site location: inferred from neighbouring sites
*wR*(*F*^2^) = 0.214	H-atom parameters constrained
*S* = 1.17	*w* = 1/[σ^2^(*F*_o_^2^) + (0.071*P*)^2^ + 2.358*P*] where *P* = (*F*_o_^2^ + 2*F*_c_^2^)/3
3306 reflections	(Δ/σ)_max_ < 0.001
271 parameters	Δρ_max_ = 0.64 e Å^−^^3^
0 restraints	Δρ_min_ = −0.73 e Å^−^^3^

### Special details

**Table:**

Geometry. All s.u.'s (except the s.u. in the dihedral angle between two l.s. planes) are estimated using the full covariance matrix. The cell s.u.'s are taken into account individually in the estimation of s.u.'s in distances, angles and torsion angles; correlations between s.u.'s in cell parameters are only used when they are defined by crystal symmetry. An approximate (isotropic) treatment of cell s.u.'s is used for estimating s.u.'s involving l.s. planes.
Refinement. Refinement of *F*^2^ against ALL reflections. The weighted *R*-factor w*R* and goodness of fit *S* are based on *F*^2^, conventional *R*-factors *R* are based on *F*, with *F* set to zero for negative *F*^2^. The threshold expression of *F*^2^ > 2σ(*F*^2^) is used only for calculating *R*-factors(gt) etc. and is not relevant to the choice of reflections for refinement. *R*-factors based on *F*^2^ are statistically about twice as large as those based on *F*, and *R*-factors based on ALL data will be even larger.

### Fractional atomic coordinates and isotropic or equivalent isotropic displacement parameters (Å^2^)

**Table:**

	*x*	*y*	*z*	*U*_iso_*/*U*_eq_	Occ. (<1)
Ca1	0.23787 (12)	0.95355 (10)	0.48080 (8)	0.0330 (3)	
N1	0.1493 (6)	0.8835 (5)	0.0638 (4)	0.0459 (12)	
F1	0.1676 (5)	1.4460 (3)	0.4314 (4)	0.0647 (11)	
O1	0.2997 (5)	0.8935 (4)	0.3020 (3)	0.0428 (9)	
H1B	0.3916	0.9137	0.2857	0.064*	
H1A	0.2274	0.8545	0.2473	0.064*	
O2	0.1035 (5)	0.7532 (4)	0.5270 (4)	0.0517 (11)	
H2B	0.1394	0.6793	0.5299	0.078*	
H2A	0.0534	0.7759	0.5762	0.078*	
O3	0.4861 (4)	1.1408 (4)	0.4636 (3)	0.0380 (9)	
O4	0.2084 (4)	1.1781 (4)	0.4336 (3)	0.0436 (10)	
O5	0.3472 (5)	1.0424 (4)	0.6789 (3)	0.0507 (11)	
O6	0.0613 (4)	1.0541 (4)	0.6240 (3)	0.0375 (9)	
C1	0.2246 (8)	0.7775 (7)	0.0316 (5)	0.0537 (15)	
H1	0.3002	0.7371	0.0879	0.064*	
C2	0.1969 (9)	0.7248 (7)	−0.0800 (6)	0.0586 (17)	
H2	0.2531	0.6515	−0.0983	0.070*	
C3	0.0851 (9)	0.7826 (7)	−0.1626 (5)	0.0593 (17)	
H3	0.0631	0.7486	−0.2386	0.071*	
C4	0.0047 (9)	0.8916 (6)	−0.1334 (5)	0.0529 (15)	
H4	−0.0718	0.9320	−0.1893	0.063*	
C5	0.0395 (7)	0.9410 (5)	−0.0186 (4)	0.0392 (12)	
C6	0.3995 (6)	1.3357 (5)	0.3811 (4)	0.0336 (11)	
C7	0.5394 (7)	1.3466 (6)	0.3307 (5)	0.0419 (13)	
H7	0.6040	1.2740	0.3246	0.050*	
C8	0.5851 (8)	1.4617 (7)	0.2894 (6)	0.0552 (16)	
H8	0.6800	1.4661	0.2570	0.066*	
C9	0.4894 (8)	1.5710 (7)	0.2961 (5)	0.0552 (16)	
H9	0.5214	1.6488	0.2692	0.066*	
C10	0.3478 (8)	1.5643 (6)	0.3423 (5)	0.0533 (16)	
H10	0.2817	1.6364	0.3461	0.064*	
C11	0.3055 (7)	1.4467 (5)	0.3833 (5)	0.0400 (12)	
C12	0.3609 (6)	1.2121 (5)	0.4293 (4)	0.0357 (12)	
C13	0.2199 (18)	1.1634 (13)	0.7949 (10)	0.0336 (19)	0.60
C14	0.1266 (13)	1.2791 (9)	0.7976 (9)	0.0575 (19)	0.60
F2	0.0396 (13)	1.3184 (9)	0.7157 (9)	0.106 (3)	0.60
C15	0.145 (3)	1.358 (2)	0.898 (2)	0.086 (4)	0.60
H15	0.0837	1.4337	0.8966	0.103*	0.60
C16	0.245 (4)	1.331 (3)	0.996 (2)	0.098 (5)	0.60
H16	0.2534	1.3858	1.0627	0.117*	0.60
C17	0.339 (4)	1.220 (3)	0.9984 (15)	0.077 (4)	0.60
H17	0.4103	1.2005	1.0676	0.093*	0.60
C18	0.3310 (17)	1.1353 (13)	0.8991 (9)	0.046 (2)	0.60
H18	0.3973	1.0624	0.9022	0.056*	0.60
C13'	0.210 (2)	1.1874 (17)	0.8095 (12)	0.0336 (19)	0.40
C14'	0.103 (3)	1.292 (2)	0.7984 (19)	0.0575 (19)	0.40
H14A	0.0297	1.3032	0.7277	0.069*	0.40
C15'	0.105 (4)	1.379 (2)	0.893 (2)	0.086 (4)	0.40
H15A	0.0335	1.4483	0.8857	0.103*	0.40
C16'	0.215 (7)	1.361 (4)	0.999 (2)	0.098 (5)	0.40
H16A	0.2165	1.4195	1.0621	0.117*	0.40
C17'	0.322 (5)	1.257 (3)	1.0098 (16)	0.077 (4)	0.40
H17A	0.3957	1.2456	1.0805	0.093*	0.40
C18'	0.3201 (18)	1.1702 (12)	0.9152 (10)	0.046 (2)	0.40
F2'	0.4186 (11)	1.0508 (9)	0.9175 (6)	0.053 (2)	0.40
C19	0.2072 (6)	1.0851 (5)	0.6954 (4)	0.0365 (12)	

### Atomic displacement parameters (Å^2^)

**Table:**

	*U*^11^	*U*^22^	*U*^33^	*U*^12^	*U*^13^	*U*^23^
Ca1	0.0245 (5)	0.0387 (6)	0.0370 (6)	0.0046 (4)	0.0087 (4)	0.0071 (4)
N1	0.042 (3)	0.055 (3)	0.040 (3)	0.005 (2)	0.009 (2)	0.006 (2)
F1	0.055 (2)	0.047 (2)	0.110 (3)	0.0161 (16)	0.048 (2)	0.019 (2)
O1	0.0347 (19)	0.059 (3)	0.0338 (19)	0.0002 (17)	0.0097 (16)	−0.0018 (17)
O2	0.039 (2)	0.043 (2)	0.085 (3)	0.0101 (17)	0.031 (2)	0.023 (2)
O3	0.0280 (17)	0.044 (2)	0.045 (2)	0.0104 (16)	0.0109 (16)	0.0129 (17)
O4	0.0304 (18)	0.036 (2)	0.071 (3)	0.0020 (15)	0.0215 (18)	0.0185 (19)
O5	0.0291 (18)	0.078 (3)	0.046 (2)	0.0059 (19)	0.0136 (17)	−0.005 (2)
O6	0.0214 (16)	0.048 (2)	0.041 (2)	0.0007 (15)	0.0059 (15)	0.0017 (16)
C1	0.050 (3)	0.063 (4)	0.050 (3)	0.012 (3)	0.012 (3)	0.011 (3)
C2	0.063 (4)	0.061 (4)	0.056 (4)	0.014 (3)	0.021 (3)	0.002 (3)
C3	0.074 (4)	0.060 (4)	0.043 (3)	0.007 (3)	0.015 (3)	−0.005 (3)
C4	0.071 (4)	0.048 (4)	0.038 (3)	0.002 (3)	0.012 (3)	0.002 (3)
C5	0.036 (3)	0.045 (3)	0.035 (3)	−0.002 (2)	0.009 (2)	0.002 (2)
C6	0.030 (2)	0.032 (3)	0.039 (3)	0.001 (2)	0.008 (2)	0.008 (2)
C7	0.038 (3)	0.042 (3)	0.050 (3)	0.006 (2)	0.015 (3)	0.014 (3)
C8	0.052 (3)	0.062 (4)	0.059 (4)	0.001 (3)	0.025 (3)	0.019 (3)
C9	0.060 (4)	0.057 (4)	0.055 (4)	0.000 (3)	0.023 (3)	0.018 (3)
C10	0.056 (4)	0.036 (3)	0.065 (4)	0.009 (3)	0.007 (3)	0.017 (3)
C11	0.035 (3)	0.042 (3)	0.049 (3)	0.004 (2)	0.017 (2)	0.009 (2)
C12	0.024 (2)	0.047 (3)	0.034 (3)	0.002 (2)	0.003 (2)	0.005 (2)
C13	0.033 (3)	0.026 (5)	0.044 (4)	0.004 (3)	0.022 (3)	−0.018 (4)
C14	0.049 (4)	0.057 (5)	0.078 (5)	0.003 (4)	0.037 (4)	0.008 (4)
F2	0.101 (6)	0.097 (7)	0.122 (7)	0.025 (5)	0.026 (6)	0.016 (6)
C15	0.073 (11)	0.059 (7)	0.141 (9)	−0.013 (5)	0.078 (8)	−0.042 (7)
C16	0.088 (14)	0.095 (14)	0.114 (8)	−0.034 (8)	0.068 (8)	−0.075 (8)
C17	0.066 (8)	0.110 (17)	0.048 (5)	−0.032 (8)	0.023 (5)	−0.044 (6)
C18	0.050 (4)	0.060 (7)	0.025 (4)	−0.017 (5)	0.014 (3)	−0.027 (4)
C13'	0.033 (3)	0.026 (5)	0.044 (4)	0.004 (3)	0.022 (3)	−0.018 (4)
C14'	0.049 (4)	0.057 (5)	0.078 (5)	0.003 (4)	0.037 (4)	0.008 (4)
C15'	0.073 (11)	0.059 (7)	0.141 (9)	−0.013 (5)	0.078 (8)	−0.042 (7)
C16'	0.088 (14)	0.095 (14)	0.114 (8)	−0.034 (8)	0.068 (8)	−0.075 (8)
C17'	0.066 (8)	0.110 (17)	0.048 (5)	−0.032 (8)	0.023 (5)	−0.044 (6)
C18'	0.050 (4)	0.060 (7)	0.025 (4)	−0.017 (5)	0.014 (3)	−0.027 (4)
F2'	0.050 (5)	0.070 (6)	0.035 (4)	0.017 (4)	0.001 (4)	0.016 (4)
C19	0.032 (3)	0.039 (3)	0.040 (3)	0.002 (2)	0.011 (2)	0.007 (2)

### Geometric parameters (Å, °)

**Table:**

Ca1—O1	2.381 (4)	C6—C11	1.390 (7)
Ca1—O6^i^	2.386 (3)	C6—C7	1.394 (7)
Ca1—O3^ii^	2.396 (3)	C6—C12	1.483 (7)
Ca1—O4	2.418 (4)	C7—C8	1.380 (8)
Ca1—O2	2.426 (4)	C7—H7	0.9300
Ca1—O5	2.427 (4)	C8—C9	1.388 (9)
Ca1—O6	2.662 (4)	C8—H8	0.9300
Ca1—O3	2.726 (4)	C9—C10	1.372 (9)
Ca1—C19	2.904 (5)	C9—H9	0.9300
Ca1—C12	2.935 (6)	C10—C11	1.393 (8)
Ca1—Ca1^i^	4.054 (2)	C10—H10	0.9300
Ca1—Ca1^ii^	4.106 (2)	C13—C19	1.373 (13)
Ca1—H2A	2.7683	C13—C18	1.411 (14)
N1—C1	1.338 (8)	C13—C14	1.428 (11)
N1—C5	1.349 (7)	C14—F2	1.1740
F1—C11	1.358 (6)	C14—C15	1.380 (18)
O1—H1B	0.8200	C15—C16	1.32 (2)
O1—H1A	0.8200	C15—H15	0.9300
O2—H2B	0.8200	C16—C17	1.39 (2)
O2—H2A	0.8200	C16—H16	0.9300
O3—C12	1.267 (6)	C17—C18	1.410 (15)
O3—Ca1^ii^	2.396 (3)	C17—H17	0.9300
O4—C12	1.253 (6)	C18—H18	0.9300
O5—C19	1.266 (6)	C13'—C14'	1.3900
O6—C19	1.266 (6)	C13'—C18'	1.3900
O6—Ca1^i^	2.386 (3)	C13'—C19	1.660 (17)
C1—C2	1.376 (9)	C14'—C15'	1.3900
C1—H1	0.9300	C14'—H14A	0.9300
C2—C3	1.360 (9)	C15'—C16'	1.3900
C2—H2	0.9300	C15'—H15A	0.9300
C3—C4	1.374 (9)	C16'—C17'	1.3900
C3—H3	0.9300	C16'—H16A	0.9300
C4—C5	1.399 (8)	C17'—C18'	1.3900
C4—H4	0.9300	C17'—H17A	0.9300
C5—C5^iii^	1.474 (11)	C18'—F2'	1.4796
			
O1—Ca1—O6^i^	86.09 (12)	C19—O5—Ca1	98.8 (3)
O1—Ca1—O3^ii^	77.46 (13)	C19—O6—Ca1^i^	163.8 (3)
O6^i^—Ca1—O3^ii^	152.43 (14)	C19—O6—Ca1	87.8 (3)
O1—Ca1—O4	90.21 (14)	Ca1^i^—O6—Ca1	106.69 (13)
O6^i^—Ca1—O4	77.91 (13)	N1—C1—C2	123.9 (6)
O3^ii^—Ca1—O4	123.52 (13)	N1—C1—H1	118.0
O1—Ca1—O2	104.41 (15)	C2—C1—H1	118.0
O6^i^—Ca1—O2	75.11 (13)	C3—C2—C1	118.3 (6)
O3^ii^—Ca1—O2	87.62 (13)	C3—C2—H2	120.8
O4—Ca1—O2	148.15 (13)	C1—C2—H2	120.8
O1—Ca1—O5	147.97 (13)	C2—C3—C4	119.8 (6)
O6^i^—Ca1—O5	124.39 (13)	C2—C3—H3	120.1
O3^ii^—Ca1—O5	77.36 (13)	C4—C3—H3	120.1
O4—Ca1—O5	87.44 (15)	C3—C4—C5	119.1 (6)
O2—Ca1—O5	93.99 (15)	C3—C4—H4	120.4
O1—Ca1—O6	157.37 (12)	C5—C4—H4	120.4
O6^i^—Ca1—O6	73.31 (13)	N1—C5—C4	121.3 (5)
O3^ii^—Ca1—O6	125.15 (12)	N1—C5—C5^iii^	116.6 (6)
O4—Ca1—O6	76.64 (12)	C4—C5—C5^iii^	122.1 (6)
O2—Ca1—O6	79.65 (13)	C11—C6—C7	115.6 (5)
O5—Ca1—O6	51.10 (11)	C11—C6—C12	124.3 (5)
O1—Ca1—O3	75.15 (12)	C7—C6—C12	120.2 (5)
O6^i^—Ca1—O3	123.49 (12)	C8—C7—C6	122.1 (5)
O3^ii^—Ca1—O3	73.59 (13)	C8—C7—H7	118.9
O4—Ca1—O3	50.07 (11)	C6—C7—H7	118.9
O2—Ca1—O3	160.92 (12)	C7—C8—C9	120.0 (6)
O5—Ca1—O3	78.98 (13)	C7—C8—H8	120.0
O6—Ca1—O3	108.30 (12)	C9—C8—H8	120.0
O1—Ca1—C19	166.43 (14)	C10—C9—C8	120.1 (6)
O6^i^—Ca1—C19	98.95 (14)	C10—C9—H9	119.9
O3^ii^—Ca1—C19	102.15 (14)	C8—C9—H9	119.9
O4—Ca1—C19	78.65 (14)	C9—C10—C11	118.4 (6)
O2—Ca1—C19	89.09 (15)	C9—C10—H10	120.8
O5—Ca1—C19	25.52 (13)	C11—C10—H10	120.8
O6—Ca1—C19	25.84 (12)	F1—C11—C6	120.0 (5)
O3—Ca1—C19	91.65 (13)	F1—C11—C10	116.3 (5)
O1—Ca1—C12	80.01 (14)	C6—C11—C10	123.7 (5)
O6^i^—Ca1—C12	99.63 (13)	O4—C12—O3	121.1 (5)
O3^ii^—Ca1—C12	99.11 (13)	O4—C12—C6	120.9 (4)
O4—Ca1—C12	24.73 (12)	O3—C12—C6	118.0 (4)
O2—Ca1—C12	172.69 (14)	O4—C12—Ca1	53.8 (3)
O5—Ca1—C12	84.77 (15)	O3—C12—Ca1	68.0 (3)
O6—Ca1—C12	94.05 (13)	C6—C12—Ca1	168.8 (3)
O3—Ca1—C12	25.52 (11)	C19—C13—C18	121.3 (9)
C19—Ca1—C12	86.70 (15)	C19—C13—C14	121.9 (9)
O1—Ca1—Ca1^i^	124.48 (10)	C18—C13—C14	116.8 (9)
O6^i^—Ca1—Ca1^i^	38.98 (9)	F2—C14—C15	115.4 (10)
O3^ii^—Ca1—Ca1^i^	154.16 (10)	F2—C14—C13	123.5 (5)
O4—Ca1—Ca1^i^	74.03 (9)	C15—C14—C13	120.9 (12)
O2—Ca1—Ca1^i^	74.38 (9)	C16—C15—C14	122.7 (14)
O5—Ca1—Ca1^i^	85.42 (9)	C16—C15—H15	118.7
O6—Ca1—Ca1^i^	34.33 (7)	C14—C15—H15	118.7
O3—Ca1—Ca1^i^	122.07 (9)	C15—C16—C17	118.8 (13)
C19—Ca1—Ca1^i^	60.03 (10)	C15—C16—H16	120.6
C12—Ca1—Ca1^i^	98.33 (11)	C17—C16—H16	120.6
O1—Ca1—Ca1^ii^	72.73 (9)	C16—C17—C18	122.1 (15)
O6^i^—Ca1—Ca1^ii^	152.07 (10)	C16—C17—H17	118.9
O3^ii^—Ca1—Ca1^ii^	39.55 (9)	C18—C17—H17	118.9
O4—Ca1—Ca1^ii^	84.04 (9)	C17—C18—C13	118.6 (12)
O2—Ca1—Ca1^ii^	127.10 (10)	C17—C18—H18	120.7
O5—Ca1—Ca1^ii^	75.25 (9)	C13—C18—H18	120.7
O6—Ca1—Ca1^ii^	122.98 (9)	C14'—C13'—C18'	120.0
O3—Ca1—Ca1^ii^	34.04 (7)	C14'—C13'—C19	119.4 (5)
C19—Ca1—Ca1^ii^	98.16 (11)	C18'—C13'—C19	120.6 (5)
C12—Ca1—Ca1^ii^	59.56 (10)	C13'—C14'—C15'	120.0
Ca1^i^—Ca1—Ca1^ii^	151.36 (6)	C13'—C14'—H14A	120.0
O1—Ca1—H2A	120.1	C15'—C14'—H14A	120.0
O6^i^—Ca1—H2A	73.1	C16'—C15'—C14'	120.0
O3^ii^—Ca1—H2A	96.2	C16'—C15'—H15A	120.0
O4—Ca1—H2A	135.5	C14'—C15'—H15A	120.0
O2—Ca1—H2A	16.5	C17'—C16'—C15'	120.0
O5—Ca1—H2A	82.1	C17'—C16'—H16A	120.0
O6—Ca1—H2A	63.2	C15'—C16'—H16A	120.0
O3—Ca1—H2A	160.0	C16'—C17'—C18'	120.0
C19—Ca1—H2A	73.5	C16'—C17'—H17A	120.0
C12—Ca1—H2A	157.2	C18'—C17'—H17A	120.0
Ca1^i^—Ca1—H2A	62.1	C17'—C18'—C13'	120.0
Ca1^ii^—Ca1—H2A	133.4	C17'—C18'—F2'	125.0
C1—N1—C5	117.5 (5)	C13'—C18'—F2'	114.8
Ca1—O1—H1B	124.9	O5—C19—O6	121.1 (5)
Ca1—O1—H1A	123.3	O5—C19—C13	117.2 (7)
H1B—O1—H1A	111.6	O6—C19—C13	121.7 (7)
Ca1—O2—H2B	129.3	O5—C19—C13'	120.8 (7)
Ca1—O2—H2A	106.1	O6—C19—C13'	118.0 (7)
H2B—O2—H2A	115.3	C13—C19—C13'	4.8 (6)
C12—O3—Ca1^ii^	167.0 (3)	O5—C19—Ca1	55.7 (3)
C12—O3—Ca1	86.5 (3)	O6—C19—Ca1	66.4 (3)
Ca1^ii^—O3—Ca1	106.41 (13)	C13—C19—Ca1	168.7 (8)
C12—O4—Ca1	101.4 (3)	C13'—C19—Ca1	167.9 (8)

Symmetry codes: (i) −*x*, −*y*+2, −*z*+1; (ii) −*x*+1, −*y*+2, −*z*+1; (iii) −*x*, −*y*+2, −*z*.

### Hydrogen-bond geometry (Å, °)

**Table:**

*D*—H···*A*	*D*—H	H···*A*	*D*···*A*	*D*—H···*A*
O1—H1A···N1	0.82	2.21	2.835 (5)	133
O1—H1B···O5^ii^	0.82	2.02	2.782 (5)	154
O2—H2A···O4^i^	0.82	2.12	2.741 (5)	132
O2—H2B···F1^iv^	0.82	2.62	3.363 (5)	151
C7—H7···O2^ii^	0.93	2.60	3.189 (7)	122
C10—H10···F2^v^	0.93	2.54	3.281 (2)	136

Symmetry codes:  (ii) −*x*+1, −*y*+2, −*z*+1; (i) −*x*, −*y*+2, −*z*+1; (iv) *x*, *y*−1, *z*; (v) −*x*, −*y*+3, −*z*+1.
